# Trends and treatment outcomes of drug resistant tuberculosis in Limpopo Province, South Africa (2011–2019): A Retrospective Study

**DOI:** 10.1371/journal.pone.0335600

**Published:** 2025-11-18

**Authors:** Kabelo Gabriel Kaapu, Ivy Rukasha

**Affiliations:** 1 Division of Medical Microbiology, Department of Pathology, School of Medicine, University of Limpopo, Limpopo, South Africa; 2 Department of Medical Microbiology, National Health Laboratory Service, Polokwane, Limpopo, South Africa; University of Washington Department of Global Health, UNITED STATES OF AMERICA

## Abstract

**Background:**

Drug-resistant tuberculosis (DR-TB) continues to threaten TB control efforts in South Africa, particularly in resource-limited provinces such as Limpopo. This study evaluated trends in DR-TB and evaluated treatment outcomes and predictors of unfavorable outcomes from 2011 to 2019.

**Methods:**

We conducted a retrospective cross-sectional study using data from 3,528 patients with DR-TB recorded in the Limpopo electronic registry (EDRWeb.net). Descriptive statistics characterized the demographics of the patients and the types of resistance. The associations between variables and outcomes were tested using chi-square analysis and binary logistic regression identified independent predictors of unfavorable treatment outcomes. The study period was stratified into pre-bedaquiline (BDQ) (2011–2015) and post-BDQ (2016–2019) eras to assess the impact of treatment.

**Results:**

Rifampicin-resistant TB (RR-TB) (61.7%) and multidrug-resistant TB (MDR-TB) (32.5%) were the most common. Overall, the success of the treatment was 59.0%, increasing from 54.1% in the pre-BDQ era to 65.3% after BDQ. XDR-TB had the lowest success rate (31.3%). In multivariate analysis, male sex (aOR = 1.12; 95% CI: 1.00–1.27), HIV positivity (aOR = 1.28; 95% CI: 1.11–1.47), age ≥ 35 years (aOR = 2.01; 95% CI: 1.08–3.76), and XDR-TB (aOR = 3.05; 95% CI: 1.65–5.65) were independently associated with unfavorable outcome.

**Conclusion:**

Treatment outcomes for DR-TB in Limpopo improved following the introduction of BDQ and shorter all-oral regimens but remain suboptimal, particularly among XDR-TB and HIV co-infected patients. Strengthening TB/HIV integration, expanding access to new drug regimens, and enhancing early diagnosis are essential to improve outcomes in rural high-burden settings.

## 1 Introduction

Tuberculosis (TB) remains a major global health challenge, with an estimated one in four people infected and over 10 million new cases annually. Despite efforts by the World Health Organization (WHO) – including the DOTS, Stop TB, and End TB strategies – progress remains limited, particularly in high-burden countries like South Africa, which has been consistently reported as one of the five countries with the greatest burdens of TB worldwide [1–4]. WHO measures an estimated 10.8 million people developed TB disease and approximately 1.25 million died from it in 2023, including 161,000 individuals living with HIV [5].

The emergence of drug-resistant TB (DR-TB), especially MDR-TB and extensively drug-resistant (XDR-TB), has further complicated TB control efforts. These strains are resistant to key first-line drugs such as rifampicin and isoniazid and require prolonged, more toxic, and costly treatments. Limpopo Province, a predominantly rural region of South Africa, faces unique challenges: higher than average mortality rates from TB, under-resourced health infrastructure, and a high prevalence of HIV among TB patients [6,7].

The province is also a gateway for South Africa to the rest of the African countries and has eight border positions that connect the country to Botswana, Mozambique and Zimbabwe. The frequent cross-border movement of people, particularly through the Mozambique and Zimbabwe border gates, may contribute to increased TB transmission dynamics due to population mobility and limited continuity of care across health systems. [8]. Efforts such as decentralization of care, the introduction of GeneXpert MTB/RIF (2011), and access to new drugs like BDQ (since 2015) aimed to improve diagnosis and outcomes. However, the burden of DR-TB remains high and treatment success rates continue to fall short of desired outcomes [6,9,10].

This study analyzes nine years of provincial data (2011–2019) to assess DR-TB trends and treatment outcomes in Limpopo, identifying risk factors for unfavorable outcomes. The findings aim to inform localized TB control strategies in similarly resource-limited, high-burden settings.

## 2. Methodology

### 2.1 Ethics approval

The study was approved by the Turfloop Research Ethics Committee (TREC) of the University of Limpopo (TREC/150/2023: IR). All clinical data were obtained following ethical guidelines, such as obtaining permission from the Limpopo Department of Health provincial ethics committee through the provincial health research database (LP-2020-10-008).

### 2.2 Study design

This study used a retrospective cross-sectional design using routinely collected data from the Limpopo Province Electronic DR-TB database (EDRWeb.net). The analysis included all patients diagnosed with laboratory-confirmed DR-TB between 1 January 2011 and 31 December 2019, whose treatment results were recorded. The study aimed to describe temporal trends in DR-TB cases and evaluate associations between demographic/clinical factors and treatment outcomes. Data were extracted on patient age, sex, HIV status, type of TB, resistance profile, treatment history, and final outcomes (e.g., cure, death, treatment failure or loss to follow-up). Statistical analysis included descriptive statistics, chi-square testing, and binary logistic regression to identify independent predictors of unfavorable outcomes.

### 2.3 Study site

Limpopo province is the fifth largest province in northern South Africa. The population of the province contributes about 10.6% of the population of South Africa (over 62 million, as of 2022), with an estimated population of 6,572,720; this makes Limpopo the fifth most populated province in the country [11,12]. Limpopo is one of the provinces most affected by TB, and the number of TB infections increased from 11,897 in 2005–22,158 in 2011 [13]. Limpopo Province has a high HIV burden and is predominantly rural, with significant barriers to access to healthcare and support for adherence. These contextual factors may influence treatment outcomes, particularly for patients with co-existing DR-TB and HIV [11].

### 2.4 Data collection

This was a retrospective cross-sectional record-based review of routinely collected data for new TB patients registered in the Limpopo Provincial electronic TB database (EDRWeb.net) between 01 January 2011 and 31 December 2019. EDRWeb.net is the software used to capture TB data in South Africa. Tuberculosis data in Limpopo Province were recorded in a paper-based TB registry at the primary health care level. These data are then captured on EDRWeb.net at the subdistrict level and sent to the Provincial Department of Health, where they are aggregated. The study population was defined as all patients with DR-TB whose treatment outcome was recorded between January 2011 and December 2019 on EDRWeb. Patients who were still on treatment were also considered. Patients may have GeneXpert, smear, culture-positive/negative pulmonary TB (PTB), or extrapulmonary TB (EPTB) results. All age groups were included in this analysis.

### 2.5 Treatment outcome

Treatment outcomes were classified according to WHO definitions as favorable (cured or completed treatment) and unfavorable (treatment failure, death, or loss to follow-up) [14]. Favorable outcomes included patients who were cured or had completed treatment. A patient was considered cured if they had bacteriologically confirmed TB at the beginning of treatment and completed treatment as recommended by the national policy with evidence of bacteriological response and no evidence of failure. Treatment completed refers to a patient who completed treatment as recommended by the national policy whose outcome does not meet the definition for cure or treatment failure.

Unfavorable outcomes included failure of treatment, death, and loss of follow-up (LTFU). Treatment failure refers to patient whose treatment regimen needed to be terminated or permanently changed to a new regimen or treatment strategy. Death refers to a patient who died before starting treatment or during the course of treatment. Loss to follow-up was defined as patient who did not start treatment or whose treatment was interrupted for 2 consecutive months or more.

### 2.6 Inclusion and exclusion criteria

**Fig 1:**
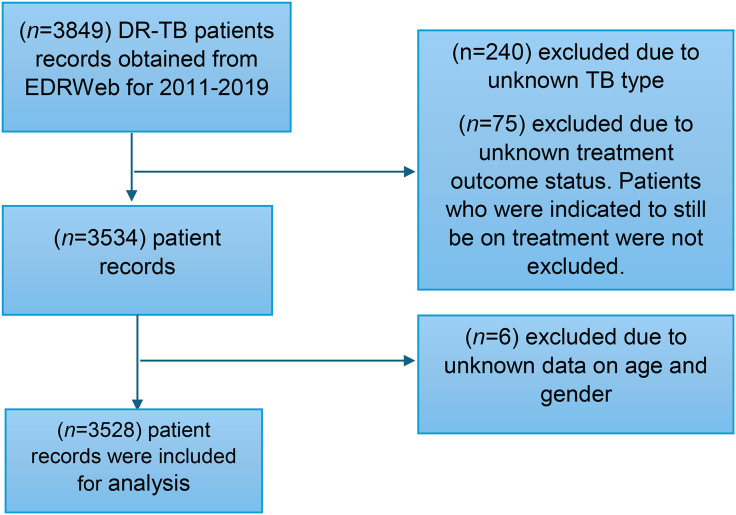
Flow diagram 1: Inclusion and exclusion criteria.

Inclusion criteria comprised all patients diagnosed with laboratory-confirmed DR-TB (pulmonary and extrapulmonary) between January 2011 and December 2019, with complete demographic, clinical and treatment outcome data recorded in the EDRWeb system. Exclusion criteria included patients without recorded treatment outcomes, incomplete data entries, and unknown type of DR-TB. However, patients who were indicated to still be on treatment were not excluded (Fig 1).

### 2.7 Data analysis

The data were cleaned and coded in Microsoft Excel and then exported to IBM SPSS Statistics version 27.0 for analysis. Descriptive statistics were used to summarize demographic and clinical characteristics of the patient. Categorical variables were presented as frequencies and percentages, while continuous variables were reported as means with standard deviation (SD). Associations between categorical variables were assessed using the Chi-square test. When the assumption of expected cell counts ≥5 was not met, Fisher’s exact test was used instead. A p-value <0.05 was considered statistically significant. A multivariate logistic regression analysis was performed to assess independent associations between age, sex, HIV status, resistance profile, and treatment category with unfavorable treatment outcomes. Both crude and adjusted odds ratios (ORs) with 95% confidence intervals (CI) were reported. The adjusted model included variables such as sex, age group, HIV status, resistance profile, and treatment category. A two-tailed p-value <0.05 was considered statistically significant.

To evaluate the impact of newer treatment regimens (for example, BDQ), the study period was divided into two phases: 2011–2015 (pre-BDQ) and 2016–2019 (post-BDQ scale-up), allowing comparison of results before and after implementation of updated treatment protocols.

## Results

### Analysis of demographic data

A total of 3528 patients with DR-TB met the inclusion criteria for the analysis, with a mean age of 44 years (±SD = 13.14, Range 01–92 years). A slight majority of the patients (1,979; 55%) were male. The highest proportion of patients fell within the 35–44 age group (1,221; 34.6%), followed by the 25–34 age group (934; 24.6%). Age groups <1–14 years and >65 years were the least common age groups, with 12 (0.4%) and 38 (1.1%) patients, respectively (Table 1).

**Table 1:** Demographic and clinical characteristics of patients enrolled in DR-TB treatment in Limpopo from 2011–2019.

Variable	Category	RR-TB n (%)	MDR-TB n (%)	Pre-XDR-TB n (%)	XDR-TB n (%)	p-value	Total
Gender	Male	1213 (62.4%)	616 (31.7%)	81 (4.2%)	33 (1.7%)	0.147	1943
	Female	964 (60.8%)	533 (33.6%)	73 (4.6%)	15 (0.9%)		1585
Age Group	0–14	50 (66.7%)	25 (33.3%)	0 (0%)	0 (0%)	**0.006**	75
	15–24	186 (53.8%)	134 (38.7%)	22 (6.4%)	4 (1.2%)		346
	25–34	595 (63.7%)	283 (30.3%)	45 (4.8%)	11 (1.2%)		934
	35–44	765 (62.6%)	381 (31.2%)	56 (4.6%)	19 (1.6%)		1221
	45–54	309 (58.9%)	179 (34.1%)	26 (5.0%)	11 (2.1%)		525
	55–64	192 (62.5%)	109 (35.5%)	3 (1.0%)	3 (1.0%)		307
	≥65	80 (66.7%)	38 (31.7%)	2 (1.7%)	0 (0%)		120
Type of TB	Pulmonary	2159 (61.7%)	1138 (32.5%)	154 (4.4%)	47 (1.3%)	0.502	3498
	Extrapulmonary	18 (60.0%)	11 (36.7%)	0 (0%)	1 (3.3%)		30
Year	2011	75 (30.9%)	139 (57.2%)	17 (7.0%)	12 (4.9%)	**<0.0001**	243
	2012	113 (42.3%)	145 (54.3%)	5 (1.9%)	4 (1.5%)		267
	2013	269 (62.1%)	151 (34.9%)	8 (1.8%)	5 (1.2%)		433
	2014	316 (67.7%)	143 (30.6%)	7 (1.5%)	1 (0.2%)		467
	2015	379 (67.9%)	157 (28.1%)	13 (2.3%)	9 (1.6%)		558
	2016	274 (60.9%)	142 (31.5%)	28 (6.2%)	6 (1.3%)		450
	2017	252 (64.8%)	106 (27.2%)	27 (6.9%)	4 (1.0%)		389
	2018	277 (67.6%)	104 (25.4%)	24 (5.9%)	5 (1.2%)		410
	2019	222 (71.4%)	62 (19.9%)	25 (8.0%)	2 (0.6%)		311
Patient Category	New	1069 (66.8%)	437 (27.3%)	78 (4.9%)	17 (1.1%)	**<0.0001**	1601
	Relapse	504 (69.3%)	185 (25.5%)	27 (3.7%)	11 (1.5%)		727
	Treatment after failure	355 (45.1%)	389 (49.4%)	33 (4.2%)	10 (1.3%)		788
	Treatment after LTFU	169 (57.7%)	101 (34.5%)	13 (4.4%)	10 (3.4%)		293

*119 patients did not have a treatment category; p-value was determined by the chi-square test. For variables in which any expected cell count was less than 5 (e.g., Type of TB and certain Age Group categories), Fisher’s exact test was applied instead of the Chi-square test

### Analysis of DR-TB trends

**Fig 2:**
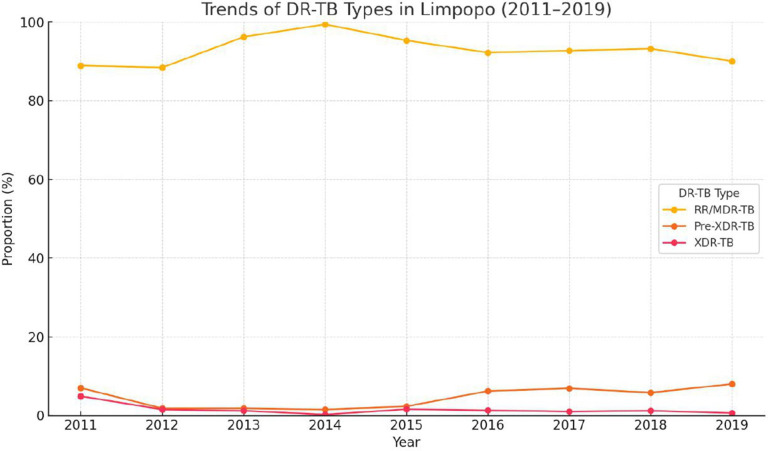
Trends of DR-TB Types in Limpopo (2011–2019).

The majority (1601; 46.9%) of DR-TB patients were new patients, followed by patients who were treated after failure (788; 23%). The highest number of patients enrolled in treatment was in 2015 (558; 15.8%), followed by 2014 (467; 13.2%), 2016 (450; 12.8%), and 2013 (433; 12.3%) (Table 1). Fig 2 presents trends in the proportions of DR-TB in Limpopo from 2011 to 2019. The data reveal that RR/MDR-TB constituted most cases of DR-TB, remaining consistently high at around >80% throughout the study period. In contrast, the proportions of pre-XDR and XDR-TB remained low and stable, consistently below 10%. While a slight potential increase in XDR-TB is observed in the later years, further research is needed to confirm its significance.

### Drug resistant TB and HIV

Of the 3,528 patients diagnosed with DR-TB, 2,394 (67.8%) were HIV positive. Among these patients with DR-TB, the proportion of HIV positive individuals was higher in females, with 75.1% co-infected. RR-TB patients constituted the majority of HIV-positive cases, representing 1,514 (63.2%) of them, as shown in Table 2.

**Table 2:** Distribution of HIV status among DR-TB patients by resistance pattern.

HIV Status	RR-TB n (%)	MDR-TB n (%)	Pre-XDR-TB n (%)	XDR-TB n (%)
Positive (n = 2394)	1514 (63.2%)	737 (30.8%)	110 (4.6%)	33 (1.4%)
Negative (n = 981)	555 (56.6%)	373 (38.0%)	37 (3.8%)	15 (1.5%)
Unknown (n = 153)	108 (70.6%)	39 (25.5%)	7 (4.6%)	0 (0%)

### Drug resistant TB treatment outcomes

**Fig 3:**
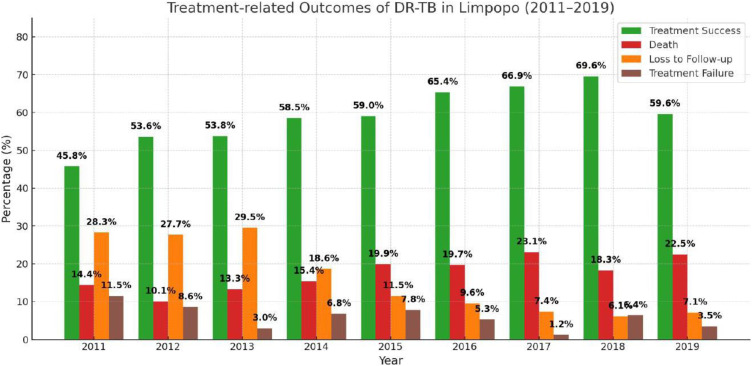
Treatment-related Outcomes of DR-TB in Limpopo, 2011–2019.

Among the 3528 patients with DR-TB, the majority (2081; 59.9%) were cured, followed by death (627; 17.7%), followed by loss of follow-up (541; 15.3%) and treatment failure was observed in (188; 5.3%) and 91 (2.6%) patients were still in treatment. Although the success of treatment improved from an average of 54.1% (2011–2015) to 65.3% (2016–2019), it remains substantially below the global target of the WHO of 90%, highlighting persistent challenges in the treatment of DR-TB in the province Fig 3.

Favorable treatment outcomes were achieved in 2081 (59.9%) patients. In particular, the loss of follow-up decreased from an average of 23.1% in the pre-BDQ era (2011–2015) to 7.6% post 2016. Similarly, treatment failure decreased from 7.5% to 3.0% over the same periods (Fig 3).

**Fig 4:**
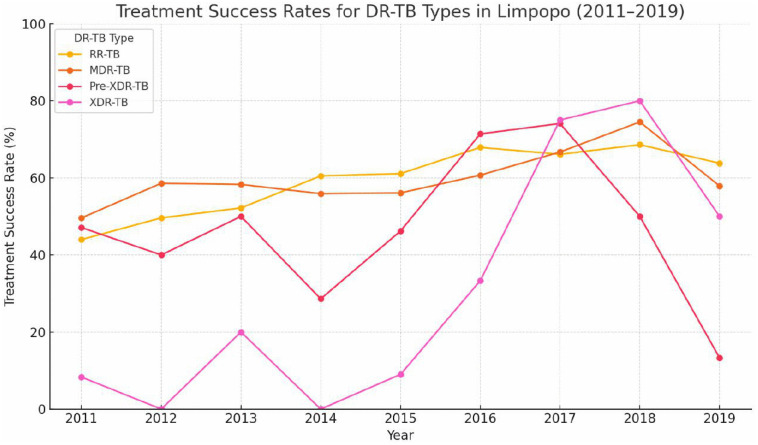
Treatment success rates for RR, MDR, pre-XDR and XDR-TB in Limpopo, 2011–2019.

Treatment success varied by resistance category, with patients with RR-TB and MDR-TB showing higher success rates of 60.3% (1,313/2,177) and 58.9% (677/1,149), respectively. On the contrary, patients with pre-XDR-TB had a lower success rate of 49.3% (76/154) and patients with XDR-TB had the poorest results, with only 15/48 (31.3%) achieving treatment success. Temporal trends indicate a gradual improvement in treatment outcomes over time. From 2011 to 2015, overall success rates remained below 60% annually, starting at 45.8% in 2011 and rising modestly to 59.0% in 2015. In particular, from 2016 onward, following the introduction and scaling up of BDQ and shorter all-oral regimens, treatment success rates consistently exceeded 65%, reaching 69.6% in 2018. While the results for XDR-TB remained suboptimal throughout, there was a marked improvement in the later years, with success rates rising from 0 to 20% before 2016–3/4 (75%) patients in 2017 and 4/5 (80%) patients in 2018, followed by a notable decline to 50% (1/2 patients) in 2019. Fig 4 shows that in the later years treatment outcomes improved, particularly for RR/MDR TB.

Table 3 shows the independent associations between demographic and clinical variables and unfavorable treatment outcomes among DR-TB patients. Adjusted ORs calculated using multivariate logistic regression controlling for gender, age group, HIV status, resistance profile, and treatment category. 154 patients had unknown HIV status and 45 had no treatment history. These were excluded from the regression model.

**Table 3:** Logistic regression analysis of independent variable associations with unfavorable treatment outcomes (n = 3528).

Variable	Unfavorable n (%)	Crude OR (95% CI)	Crude OR p value	Adjusted OR (95% CI)	Adjusted OR p-value
Gender					
Female (Ref)	582 (36.7%)	Ref		Ref	
Male	781 (40.1%)	1.16 (1.03–1.31)	**0.018**	1.12 (1.00–1.27)	**0.045**
Age Group					
<15 (Ref)	16 (21.3%)	Ref		Ref	
15–34	512 (40.0%)	2.42 (1.3–4.6)	**0.006**	2.21 (1.18–4.15)	**0.014**
≥35	790 (36.3%)	2.12 (1.1–4.1)	**0.020**	2.01 (1.08–3.76)	**0.028**
HIV Status					
Negative (Ref)	325 (33.2%)	Ref		Ref	
Positive	983 (41.1%)	1.34 (1.17–1.53)	**<0.001**	1.28 (1.11–1.47)	**<0.001**
Resistance Profile					
RR-TB (Ref)	812 (37.2%)	Ref		Ref	
MDR-TB	451 (39.2%)	1.09 (0.95–1.24)	0.210	1.06 (0.93–1.22)	0.385
Pre-XDR-TB	67 (43.5%)	1.31 (0.91–1.89)	0.145	1.22 (0.83–1.78)	0.310
XDR-TB	33 (68.8%)	3.47 (2.0–6.1)	**<0.001**	3.05 (1.65–5.65)	**<0.001**
Treatment Category					
New (Ref)	577 (36.0%)	Ref		Ref	
Retreatment	775 (41.1%)	1.24 (1.01–1.52)	**0.037**	1.18 (0.97–1.43)	0.099

Male patients had significantly higher odds of unfavorable outcomes compared to females (aOR: 1.12; 95% CI: 1.00–1.27; p = 0.045), indicating a modest increase in the odds of experiencing an unfavorable outcome.

The drug resistance pattern was strongly associated with poorer outcomes. Although RR-TB patients served as the reference group, the odds of unfavorable outcomes increased with higher resistance levels: MDR-TB (aOR: 1.06; 95% CI: 0.93–1.22), pre-XDR-TB (aOR: 1.22; 95% CI: 0.83–1.78) and XDR-TB (aOR: 3.05; 95% CI: 1.65–5.65; p < 0.001), with significantly higher odds of unfavorable outcomes among patients with XDR-TB.

HIV-positive patients had significantly higher odds of unfavorable treatment outcomes compared to HIV-negative individuals (aOR: 1.28; 95% CI: 1.11–1.47; p < 0.001), indicating that HIV status is independently associated with increased odds of an unfavorable outcome.

Age was also significantly associated with the results (p = 0.014). Using children under 15 years of age as the reference group (21.3%), patients 15–34 years of age had the highest odds of unfavorable outcomes (aOR: 2.21; 95% CI: 1.18–4.15), followed by those aged ≥35 years (aOR: 2.01; 95% CI: 1.08–3.76), compared to patients under 15 years of age.

Regarding the treatment category, retreatment cases had higher odds of unfavorable outcomes (aOR: 1.18; 95% CI: 0.97–1.43) compared to new cases of TB, although this association was not statistically significant (p = 0.099).

## Discussion

This study describes the trends and treatment results of DR-TB in Limpopo province, South Africa, from 2011 to 2019. Most of the patients (55%) were between 15 and 54 years old, the economically active group, which is more likely to contract TB due to exposure in crowded environments [15]. Men accounted for 68.8% of cases, consistent with previous studies indicating higher TB risk in men [16–18]. This is possibly due to behavioral factors such as smoking, alcohol use, and delayed healthcare-seeking [17,19–21]. Children may be underrepresented due to diagnostic challenges.

The number of people with RR/MDR-TB disease increased from 2011 to 2019, in line with the findings from other studies [22,23]. A national DR-TB survey in South Africa (2012–2014) reported a nearly double of resistance to rifampicin resistance (1.8% to 3.4%) [24]. GeneXpert MTB/RIF, introduced in 2011–2012, improved the detection of RR-TB by 30%, leading to better case identification and surveillance compared to traditional culture methods [25–29]. However, GeneXpert primarily detects resistance to rifampicin, which means that some cases may not have been fully classified as MDR-TB due to limited resistance testing of isoniazid, particularly in resource-limited settings.

Our study found that RR/ MDR-TB is the predominant form of drug resistance (~90% of patients with DR-TB), consistent with national TB prevalence surveys [6,30]. The persistent high RR/MDR-TB disease in Limpopo underscores the urgent need for a strengthened diagnostic capacity to rapidly identify drug resistance and initiate appropriate treatment.

The success rates of DR-TB treatment were low, but improved over time, increasing from 45.8% in 2011 to 69.6% in 2018. Similar trends have been reported in Gauteng (48.8%) and other South African studies (~43.4%) [31,32]. However, South Africa introduced oral drug treatment for patients with MDR-TB patients during the study period [33]. The introduction of shorter all-oral regimens may have contributed significantly to treatment success, as oral medications are more tolerable than painful injectable regimens, which were associated with poor adherence and adverse events. Shorter regimens have been reported to improve treatment results by reducing duration from 18–24 months to 6--9 months, improving adherence, and lowering LTFU rates [34–37]. This has led to higher cure rates, better patient adherence, and reduced treatment LTFU rates. Shorter regimens also reduce the infectious period, helping limit the spread of drug-resistant TB strains. Although BDQ was introduced in 2015, the transition to shorter all-oral DR-TB regimens occurred in a phased way in South Africa. In Limpopo province, implementation was gradual, with a broader acceptance beginning in 2016. This justifies the division of the analysis into pre- (2011–2015) and post-BDQ (2016–2019) periods to reflect the change in treatment policy.

Despite progress, treatment outcomes for pre-XDR and XDR-TB remained poor, although improved with the introduction of BDQ and delamanid. These drugs increased cure rates from ~30% to over 60–70% [36,38,39], transforming XDR-TB from a highly fatal disease into a more manageable condition. Their inclusion in WHO-recommended regimens highlights their role in improving treatment success, but access barriers remain, particularly in rural settings. Most of the patients with XDR-TB in this study were previously exposed to second-line drugs, increasing their risk of failure of treatment and further resistance. Enhanced support and targeted strategies are needed to overcome these challenges and ensure that patients benefit from these advancements in TB treatment.

Given the high HIV burden in Limpopo, we next examine its interaction with DR-TB. HIV co-infection was a major factor affecting results, with 67.8% of the study population being HIV positive. HIV is a well-established risk factor for poor DR-TB outcomes, even among patients receiving antiretroviral therapy (ART) [40–42]. Poor treatment success among patients with pre-XDR/ XDR-TB may partly reflect challenges in ensuring adherence to TB and ART regimens in co-infected individuals. Strengthening integrated care and patient support remains essential to improve outcomes in this vulnerable group. [43,44]. Ensuring adherence to TB and ART regimens is critical for improving outcomes in co-infected individuals, especially in a mostly rural setting like Limpopo.

An increase in mortality rates was observed during the study period. This may reflect a combination of factors, including improved case detection, late presentation, comorbidities such as HIV, and complex resistance patterns. Although HIV co-infection is known to be associated with higher mortality in DR-TB, further research is needed to understand the timing and effectiveness of ART initiation in this population. [41,42],

The findings of this study underscore the persistent burden of DR-TB in Limpopo and the critical need for stronger TB control measures. While diagnostic advancements and treatment innovations have improved detection rates and treatment success, the high number of RR/MDR-TB disease, poor outcomes in XDR-TB cases, and the strong link between HIV co-infection and mortality remain pressing challenges.

### Limitations

This study relied on routine programmatic data, which may have limitations in completeness and accuracy. Patients with missing treatment outcomes were excluded from the analysis, which may have introduced selection bias and potentially affected the generalizability of the findings. More research with comprehensive sampling strategies is needed to confirm these findings and explore additional risk factors. Furthermore, we lack data on simultaneous testing of RIF and INH, which can affect the classification of RR-TB cases. Sociodemographic data and pharmacovigilance records were also limited, preventing an analysis of factors that influence treatment outcomes. Furthermore, the definitions of pre-XDR/ XDR-TB were updated in 2021, but our study used pre-2021 classifications. The impact of BDQ, which was expanded in Limpopo only from 2016, warrants further investigation.

This study treated data from different years as independent cross-sectional snapshots, without applying longitudinal statistical methods such as Generalized Estimating Equations (GEE) or Generalized Linear Mixed Models (GLMM). This approach may not fully capture potential correlations or temporal trends over years, which could affect the robustness of observed associations. Future studies using hierarchical or longitudinal models are recommended to validate these findings.

## Conclusion

This study highlights an increasing number of drug-resistant TB cases diagnosed in Limpopo during the 2011–2019 period, probably influenced in part by the expansion of diagnostic tools such as GeneXpert. While treatment outcomes improved over time, significant challenges remain, particularly among pre-XDR and XDR-TB patients and those co-infected with HIV. These findings underscore the need for continued investment in early diagnosis, access to new TB therapies, and robust healthcare infrastructure to improve outcomes for DR-TB patients in resource-limited settings.

## Supporting information

S1 File Supporting data. (ZIP)
